# Experimental sepsis causes SERCA2 expression in white adipose tissue but not classical browning

**DOI:** 10.1038/s41598-026-60904-0

**Published:** 2026-07-03

**Authors:** Robert Tidswell, Ali Alqallaf, Joseph Harris, Jonathan Falconer, Pelin A. Golforoush, Nishkantha Arulkumaran, Anna Kleyman, David Brealey, Matthew S. Rodeheffer, Ketan Patel, Michael R. Duchen, Mervyn Singer

**Affiliations:** 1 Division of Medicine, Bloomsbury Institute of Intensive Care Medicine, University College London, London, UK; 2 School of Biological Sciences, University of Reading, Hopkins Building, Reading, UK; 3 The Hatter Cardiovascular Institute, University College London, London, UK; 4 Departments of Comparative Medicine, Cellular and Molecular Physiology and the Yale Centre of Molecular and Systems Metabolism, Yale University School of Medicine, New Haven, CT USA; 5 Department of Cell and Developmental Biology, UCL Consortium for Mitochondrial Research, University College London, London, UK

**Keywords:** Sepsis, Adipose tissue, Browning, Thermogenesis, Cachexia, Critical illness myopathy, Diseases, Medical research

## Abstract

Sepsis causes muscle wasting and cachexia but mechanisms remain unclear. Cachexia in cancer and burn injury is partly attributed to ‘browning’; where white adipose tissue (WAT) develops a catabolic, thermogenic brown adipose tissue-like phenotype. We hypothesised that sepsis-induced muscle wasting is caused by browning. 58 male Wistar rats were randomised to sham (n = 17) or experimental sepsis induced by intraperitoneal zymosan (n = 41). Tibialis anterior mass was measured on Days 3 and 14. Browning was sought using whole body and WAT respirometry, RNA-sequencing, immunoblot, thermal imaging and multi-photon microscopy of WAT. Fourteen-day mortality in rats receiving zymosan was 17%. In survivors, body mass loss peaked at day 3 and persisted to day 14 with associated tibialis anterior muscle mass loss. Zymosan peritonitis caused hypermetabolism during the late recovery phase (Days 11–14), but no difference in epididymal white adipose tissue temperature nor oxygen flux. At Day 14 transcriptomics showed inflammation but no increase in uncoupling protein (UCP)-1 at transcript or protein levels. SERCA2 protein was however increased fourfold in retroperitoneal WAT at day 14 (*p* = 0.016). Rats recovering from zymosan peritonitis developed muscle wasting and cachexia associated with WAT inflammation and whole-body hypermetabolism. No evidence of browning was seen at functional, transcriptomic or protein levels, therefore our data do not support the hypothesis of classical browning as a driver of sepsis-induced muscle wasting and cachexia. SERCA2 protein expression was however increased in retroperitoneal WAT at day 14.

**Supplementary Information:**

The online version contains supplementary material available at https://doi.org/10.1038/s41598-026-60904-0.

## Introduction

Survivors of sepsis and other critical illnesses frequently suffer a cachexia-type syndrome characterized by hypermetabolism, tissue wasting, catabolism, immune dysfunction and chronic inflammation^1–4^. Myopathy is a prominent physical complication, seen in 25–75% of survivors and associated with increased long-term morbidity and mortality^5^. The related locomotor impairment can persist for years; survivors of acute respiratory distress syndrome, a common pulmonary complication of sepsis, have markedly reduced 6-min walk distances at 1 and 5 years after their initial illness^6,7^. Mechanisms of sepsis-induced myopathy include ubiquitin-proteosome activation, a relative reduction in protein synthesis, dysregulated autophagy and reduced progenitor cells^8,9^. However, what drives the hypermetabolism, catabolism and muscle loss is less well understood.

In two inflammatory conditions akin to sepsis, burn injury and cancer, the development of cachexia has been attributed to hypermetabolism driven by heat production through browning^10–12^. Browning describes the switch of white adipose tissue (WAT) to a thermogenic phenotype (‘beige adipose tissue’) similar to brown adipose tissue (BAT). The switch is driven by β_3_-adrenergic stimulation and inflammation that stimulate de novo beige adipocyte precursors dormant within WAT, or a phenotypic switch from classical white adipocytes^13–15^. The main mechanism of heat production is by mitochondrial uncoupling where the proton gradient is ‘uncoupled’ from adenosine triphosphate production and instead released as heat^16^. This is supported by multi-loculation of lipid droplets, increased mitochondrial density and biogenesis, and increased expression of the thermogenic mitochondrial uncoupling mechanism, uncoupling protein 1 (UCP1)^16,17^. Heat is also produced by futile substrate cycles which involve two or more reactions occurring in opposite directions with no net effect other than increased oxygen consumption and ATP hydrolysis with consequent heat generation^17,18^. Calcium cycling by SERCA2 is one of the best described UCP-1 independent thermogenic mechanisms in brown and beige adipose tissue^19^.

Browning-induced thermogenesis is believed to drive hypermetabolism in excess of calorie intake, resulting in catabolism, muscle and fat loss and cachexia, among other metabolic aberrations^20–22^. In 20 patients suffering burn injury, subcutaneous adipose tissue taken at days 20–21 revealed browning, increased mitochondrial density, lipid multiloculation and expression of UCP1^11^. Blockade of hypermetabolism in the recovery phase of burn injury using the ß-adrenergic blocker, propranolol, preserved lean tissue mass in a cohort of paediatric patients^23^. In a study of patients with advanced cancer, browning was found in almost all adipose tissue samples taken from patients with cachexia^10^. However, other studies demonstrate that while adipose tissue is remodelled, UCP1-mediated browning is absent in both patients and animals with cancer cachexia and UCP1 mRNA expression is even down regulated compared to controls^24,25^.

Correspondingly, browning has been sought in sepsis. Crowell et al. found UCP1 protein in epididymal WAT from mice at day 10 of recovery from cecal ligation and puncture (CLP), and this was associated with an inability to replete lost fat mass^26^. Browning was also identified in another CLP mouse model at 18 h by UCP1 protein expression, lipid droplet budding (taken to indicate multiloculation) and increased mitochondrial density^26–28^. Of note, obese mice were protected from browning; this was suggested to explain the ‘obesity paradox’ where obese subjects have better outcomes from sepsis, including reduced muscle wasting^28^. Conversely, another study showed CLP caused induction of inflammatory genes but not thermogenic pathways in peri-renal adipose tissue^29^.

In light of the dearth of treatments for sepsis-induced myopathy and cachexia, browning in sepsis has been proffered as a potential therapeutic target. We therefore hypothesised browning in white adipose tissue drives myopathy and cachexia in the recovery phase of a rat model of experimental sepsis.

## Methods

### Animal model

Animal experiments were performed under a Home Office Project License (PPL 70/7029) and local University College London (UCL) Ethics Committee approval. Experiments were performed in accordance with relevant guidelines and regulations. Male Wistar rats of early adulthood, approximately 12–14 weeks old, (Charles River, Harlow, UK) weighing 318–411 g were randomized to either sham (n = 17) or experimental sepsis induced by zymosan peritonitis (n = 41). Animals were acclimatized in thermoneutral conditions (26 °C) for a minimum of 24 h with ad libitum access to water and Teklad feed (Envigo, Huntingdon, UK) consisting of 18% protein, fat 6%, carbohydrate 44%.

Under subcutaneous buprenorphine analgesia (6 µg/100 g body mass) and brief isoflurane anesthesia, animals in the experimental sepsis arm received an intraperitoneal injection of zymosan (40 mg/100 g body mass) (Z4250, Sigma, Castleford, UK) suspended in liquid paraffin (232-384-2, Sigma, Castleford, UK). Zymosan is an insoluble β-1,3-glucan polysaccharide derived from the cell wall of the yeast *Saccharomyces cerevisiae* which generates a more prolonged inflammatory insult compared to a bacterial infection^30^. Sham animals received identical treatment but without intraperitoneal injection.

Body mass, food intake and illness severity score were recorded in all animals daily. Stroke volume (by echocardiography, General Electric, Boston, MA) and core temperature (by rectal thermocouple, Harvard Apparatus, Holliston, MA) were measured in 6 sham and 10 zymosan peritonitis animals at baseline, 6 h and 1, 3, 7 and 14 days. Animals demonstrate biochemical and clinical recovery from day 3 but day 14 was chosen to represent the ‘extended recovery’ phase and the ultimate experimental end-point appropriate to detect browning based on; (1) experience demonstrating clinical recovery from acute zymosan peritonitis but persistent myopathy and cachexia^30^, and (2) time points in literature on browning caused by sepsis, burn and cancer^10–12,26^. Animals were culled at 1, 3, 7 and 14 days, or prematurely if they exceeded a severity score threshold which predicts impending death within 6 h. This score is reproduced in Supplemental Materials—Table 1. Blood and tissue samples were collected at the experimental end-point. Tibialis anterior was dissected at Days 3 and 14 from the hindlimbs, weighed and snap-frozen for later batched analysis. Euthanasia was performed by exsanguination by cardiac puncture under terminal anaesthesia. The study is reported in keeping with the ARRIVE guidelines^31^.

### Metabolic monitoring

Measurement of VO_2_ and VCO_2_ was performed in 4 sham and 4 zymosan peritonitis animals for 14 days after induction of experimental sepsis. Animals were placed in individual metabolic carts (Oxymax CLAMS, Columbus, OH) in thermoneutral conditions (26 °C) and acclimatized for a minimum of 48 h. Oxygen consumption and CO_2_ production were measured every 8 min for each animal, from which the respiratory exchange ratio (VCO_2_/VO_2_) was calculated.

### Thermal imaging

Epididymal WAT temperature was measured by thermal imaging at Day 14 of recovery from experimental sepsis in 9 sham and 8 zymosan peritonitis animals. Immediately after laparotomy, eWAT and liver (used to normalize temperature variation between animals) were identified and a thermal image taken (A35 camera, FLIR, West Malling, UK). Core temperature was measured simultaneously by rectal thermocouple.

### Respirometry

High resolution respirometry was performed on epididymal (eWAT) and retroperitoneal (rpWAT) white adipose tissue in an O2k respirometer with DatLab 8 software (Oroboros Instruments, Innsbruck, Austria). Chambers underwent air calibration daily. In brief, within minutes of sacrifice, eWAT (12 sham, 9 zymosan peritonitis Day-3, 9 zymosan peritonitis Day-14) and rpWAT (4 sham, 4 zymosan peritonitis Day-14) were dissected and placed in ice-cold biopsy preservation medium (see Supplemental Methods Table 2 for constituents). After removing visible connective and vascular tissue, adipose tissues were weighed and then chopped into approximate 0.5–1 mm^3^ pieces. Permeabilisation studies identified that no chemical permeabilisation was necessary (see Supplemental Materials—Fig. 2). 40–50 mg of WAT was placed into each chamber in Mir05 respiration buffer at 37 °C. Oxygen concentration was maintained between 100 and 200 nmol/ml. Following equilibration, pyruvate (final concentration 5 mM), malate (2 mM) and glutamate (10 mM) were added to stimulate mitochondrial respiratory Complex I activity, followed by succinate (10 mM) to stimulate Complex II activity. Finally, the chemical uncoupler carbonyl cyanide *p*-trifluoro-methoxyphenyl hydrazone (FCCP) was added at a pre-optimised and saturating final concentration of 2 µM to measure maximal uncoupled oxygen consumption (flux). All experiments were performed in simultaneous duplicate.

### Serum cytokines and markers of organ function

Creatinine, urea, bilirubin, albumin, alanine transaminase and creatine kinase were measured in serum taken from 5 sham animals and 5 animals at days 1 (during the acute phase), and 14 (during extended recovery phase) of zymosan peritonitis. Measurement was performed by the Chemical Pathology Department, Royal Veterinary College (Potters Bar, Herts, UK) using photoelectric colorimetry on a AU680 analyser (Beckman Coulter, Brea, CA).

Circulating cytokines were measured by a LegendPlex bead-based multiplex assay panel (BioLegend, San Diego, CA) as per manufacturer’s instructions in serum from 8 sham animals, and animals at Days 3 (n = 5), 7 (n = 7) and 14 (n = 7) of zymosan peritonitis.

### RNA sequencing

RNA was isolated from rpWAT using an adipose tissue specific isolation kit (RNeasy Lipid Tissue Mini Kit, Qiagen, Hilden, Germany) as per protocol, without optional DNA digestion. RNA was quantified by NanoDrop (Thermofisher, Oxford, UK) and quality-checked by automated electrophoresis (Bioanalyser 2100, Agilent, Cheadle, UK). RNA sequencing was performed by NovoGene (Cambridge, Cambs, UK). Detailed methods are provided in the Supplemental Materials. Raw RNA-seq data are available on NCBI Sequence Read Archive, reference: PRJNA1482020, https://www.ncbi.nlm.nih.gov/sra/PRJNA1482020.

### Western blot

Western blot was performed to measure markers of browning in epididymal and retroperitoneal white adipose tissue from sham and zymosan peritonitis animals. UCP1 was measured in epididymal tissue from sham animals and at Days 3, 7 and 14 of zymosan peritonitis. SERCA2 and UCP1 were measured in retroperitoneal WAT from sham animals and at Day 14 of zymosan peritonitis. Detailed description of methods, including extra steps taken to reduce lipid contamination of lysates are described in the Supplemental Materials. Expression of the protein of interest was normalised to alpha-tubulin and measured by ratiometric comparison.

### Multiphoton microscopy

rpWAT was dissected from sham and zymosan peritonitis animals at Day 14 and immediately placed in ice-cold biopsy preservation medium. Before imaging, rpWAT was dissected into approximately 2 × 2 × 1 mm pieces and equilibrated to 37 °C in physiological saline solution with HEPES buffer (constituents in Supplemental Materials—Table 5), with or without fluorophores as necessary. Imaging was performed on an SP8 high-resolution upright multiphoton microscope (Leica Microsystems, Milton Keynes, Bucks, UK) with a titanium sapphire laser, an environmental chamber, a 25 × 0.95 NA water dipping objective and 2 internal and 2 external HyD photomultiplier detectors. Imaging conditions were controlled using LAS-AF software (Leica Microsystems). Tissue was imaged at 37 °C within 60 min of dissection. See Supplemental Materials for detailed imaging configuration and analysis (Supplemental Materials—Table 4).

### Statistics and data display

Normally distributed data are displayed as means with error bars representing standard deviation (SD). Where data are not normally distributed, data are displayed as medians and inter-quartile range (IQR). Normality was judged using frequency plots and normality testing using the D’Agostino–Pearson normality test. Parametric (t tests, ANOVA, mixed effects models) and non-parametric (Mann–Whitney, Kruskal–Wallis) tests were used for normally and non-normally distributed data, respectively. Where small sample sizes precluded assessment of distribution, non-parametric testing was performed. Analysis of RNA sequencing detailed in Supplemental Materials.

Table 6 in the Supplemental Materials delineates experimental timepoints.

## Results

### Animals with zymosan peritonitis develop clinical features of experimental sepsis (Fig. 1)

Seven of 41 (17%) animals receiving zymosan died or were culled prematurely due to illness severity indicating organ failure consistent with a sepsis-like syndrome. Animals in the sham group remained phenotypically normal in appearance and behavior throughout the experimental period and none died. In animals receiving zymosan, illness was apparent from 2 h post-insult with piloerection, hunched posture, facial grimacing, reduced spontaneous movement, lack of interest in their surroundings, and reduced or absent food foraging. Clinical assessment revealed panting and increased respiratory rate. Peak illness occurred at 18–36 h when the modal clinical illness severity score was 2 and food intake was at its nadir. Clinical scores were significantly higher (*p* < 0.05) in zymosan peritonitis animals (AUC 5.9 (95% CI 4.2–7.6) compared to sham (AUC 0 (95% CI 0–0). Zymosan peritonitis survivors began to recover from Day 3, signalled by normalization of appearance, behavior, resumption of food intake, and no clinical features of illness. The clinical score did not differ significantly between sham and zymosan peritonitis animals between Days 8–14 (AUC 0 (95% CI 0–0) vs. 0.17 (CI 0–3.9)). Core temperature was similar between groups at 6, 24 and 72 h, and at 7 and 14 days.

**Fig. 1:**
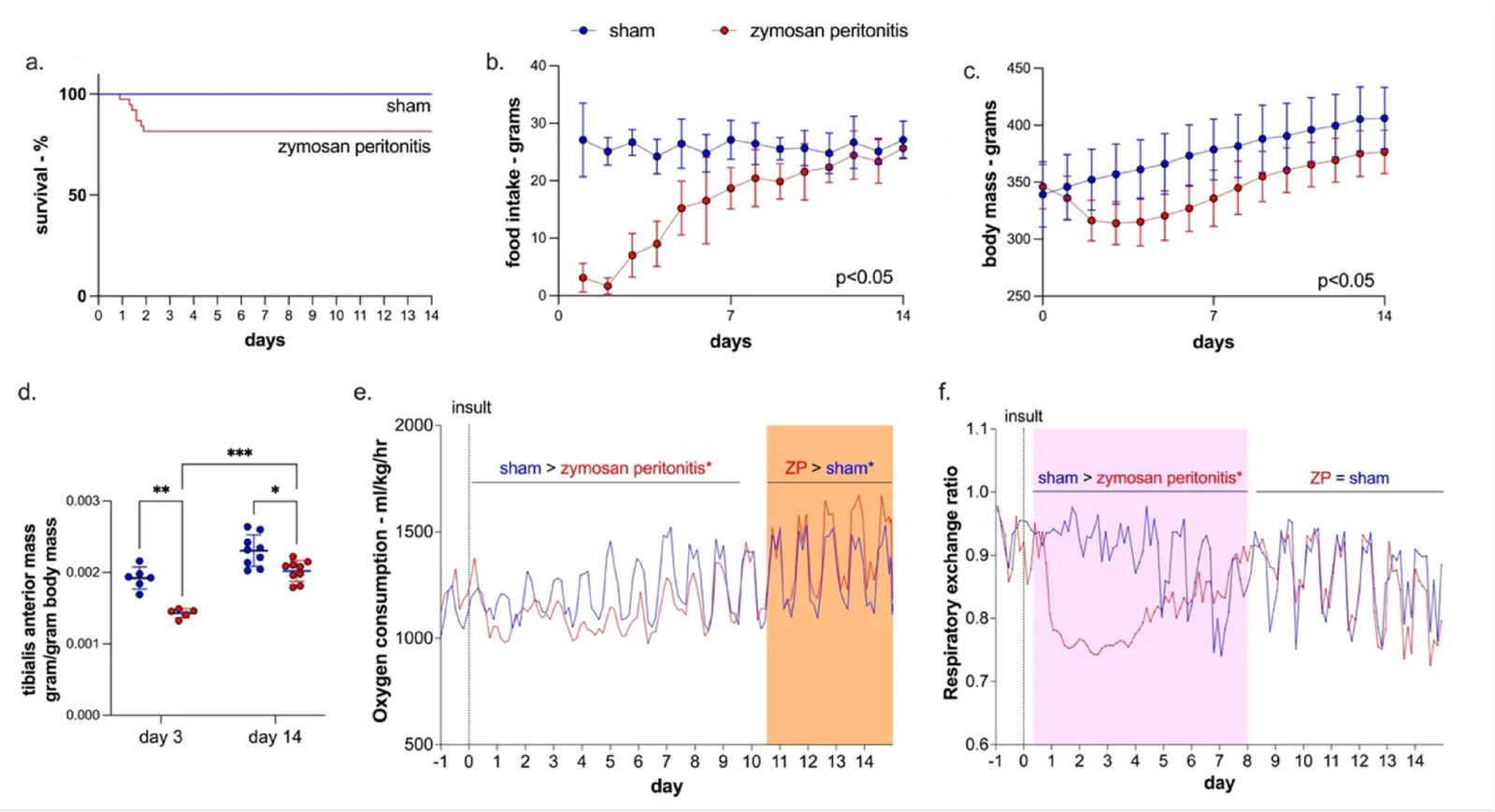
Physiological parameters of the 14-day zymosan peritonitis rat model of sepsis. (**a**) Kaplan–Meier plot of survival in sham and zymosan peritonitis animals. No sham animals died versus 7 of 41 septic animals (17%). (**b**) Daily food intake was significantly lower in zymosan peritonitis (n = 9) versus sham (n = 9) (215 g (CI 194–253) vs. AUC 336 g (CI 319–353), *p* < 0.05). Normal food intake resumed by Day 9 in the zymosan peritonitis rats. (**c**) Body mass measured daily was lower in zymosan peritonitis animals compared to sham (AUC 4797 g (CI 4690–4900) vs. 5270 g (CI 5130–5420), *p* < 0.05) and persisted to day 14. (**d**) Compared to sham, tibialis anterior (TA) deep mass normalised to body mass was reduced at Day 3 of zymosan peritonitis (*p* = 0.005) and Day 14 (*p* = 0.03). Mixed model with post-hoc analysis. (**e**) Whole organism oxygen consumption in 4 sham and 4 zymosan peritonitis animals from Day − 1 to Day 14 following the insult. Zymosan peritonitis animals showed an initial hypometabolic phase and a later hypermetabolic recovery phase (*p < 0.05, orange block indicates hypermetabolic phase). Area under the curve, unpaired t-test. (**f**) Whole organism respiratory exchange ratio (RER = VCO_2_/VO_2_) in 4 sham and 4 zymosan peritonitis animals from Day − 1 to Day 14 following the insult. RER was reduced in animals with zymosan peritonitis from days 0–8 compared to sham animals, indicating a switch to lipid metabolism. From days 9–12 the RER was equivalent between groups. The RER then fell in zymosan peritonitis animals at days 13–14 (*p < 0.05, magenta blocks indicate lipolytic phases).

In animals with zymosan peritonitis organ dysfunction was also evident by a reduction in cardiac function. Stroke volume measured at baseline, and at 6, 24, 48, 72 h and 7 days was reduced in zymosan peritonitis animals at 6 h (0.28 ± 0.04 vs. sham 0.49 ± 0.06 ml, *p* < 0.001) and 24 h (0.37 ± 0.06 vs. sham 0.48 ± 0.07 ml, *p* = 0.02).

### Animals with zymosan peritonitis develop a cachexia phenotype in the recovery phase

Sham animals ate a mean of 24 ± 3.5 g of chow per day. Zymosan peritonitis animals displayed a nadir of food intake at Day 2 with a mean intake of 1.7 ± 1.4 g. Thereafter food intake increased, reaching equivalence with sham animals by Day 10. Body mass was similar at baseline. Animal movement and activity was not formally measured however zymosan peritonitis animals were observed to display a septic phenotype which included reduced activity compared to controls. It increased in sham animals by 4.7 ± 1.8 g per day (rats of 3–4 months are still in a growth phase). Zymosan peritonitis animals displayed a nadir of body mass at Day 3 with a mean loss of 32 ± 11 g (9.2 ± 3% per day). Thereafter zymosan peritonitis animals gained weight uniformly at a mean of 6.3 ± 1.5 g per day. Zymosan peritonitis animals did not recover their sham counterparts’ mass at day 14 (406 g vs. 376 g, *p* = 0.017). Evidence of muscle wasting was present in the hindlimbs at Day 3 after the insult and persisted at Day 14: tibialis anterior mass was significantly reduced compared to sham at Day 3 (*p* = 0.005) and Day 14 (*p* = 0.03).

### Animals with zymosan peritonitis are hypometabolic in the acute phase and hypermetabolic in the extended recovery phase

Animals with zymosan peritonitis showed an initial hypometabolic phase followed by a later hypermetabolic recovery phase. Between Days 0–10, oxygen consumption was significantly lower in zymosan peritonitis animals (AUC 523 (95% CI 517–530) vs. sham 567 (562–572) litres O_2_/kg, *p* < 0.001). Between Days 11–14, zymosan peritonitis animals undergoing recovery became hypermetabolic compared to sham controls (AUC 246 (240–250) vs. sham 229 (226–233) litres O_2_/kg, *p* < 0.001).

Carbon dioxide production (VCO_2_) was lower in animals with zymosan peritonitis from Days 0–7 (AUC 392 (384–400) vs. sham 470 (465–476) litres CO_2_/kg, *p* < 0.001). From Days 8–14, CO_2_ production was equivalent between groups (AUC zymosan peritonitis 283 (280–286) l/kg vs. sham 281 (278–284) litres CO_2_/kg, *p* = 0.38) (Supplemental Materials—Fig. 1).

The respiratory exchange ratio (calculated as VCO_2_/VO_2_) was lower in animals with zymosan peritonitis from Days 0–8 (AUC 1087 (1077–1096) vs. sham 1187 (1178–1196), *p* < 0.001), indicating a switch to lipid metabolism. From Days 9–14 the RER was equivalent between groups (AUC zymosan peritonitis 748 (756–767) vs. sham 762 (733–763), *p* = 0.88).

### Zymosan peritonitis is associated with inflammation and organ dysfunction (Fig. 2)

Inflammatory markers were measured in sham animals and at Days 3, 7 and 14 of zymosan peritonitis. Serum TNFα showed a non-significant increase in zymosan peritonitis animals at Days 3 and 7. Interleukin-6, a cytokine considered crucial to browning in mice with burn injury^15^, was increased at Day 3 of zymosan peritonitis (*p* < 0.005 compared to sham) but normalized by Day 7. Monocyte chemoattractant protein-1 (MCP-1), also reported to be involved in browning and adipose-muscle crosstalk^32,33^, was elevated at Day 7 of zymosan peritonitis compared to sham (*p* < 0.05).

**Fig. 2:**
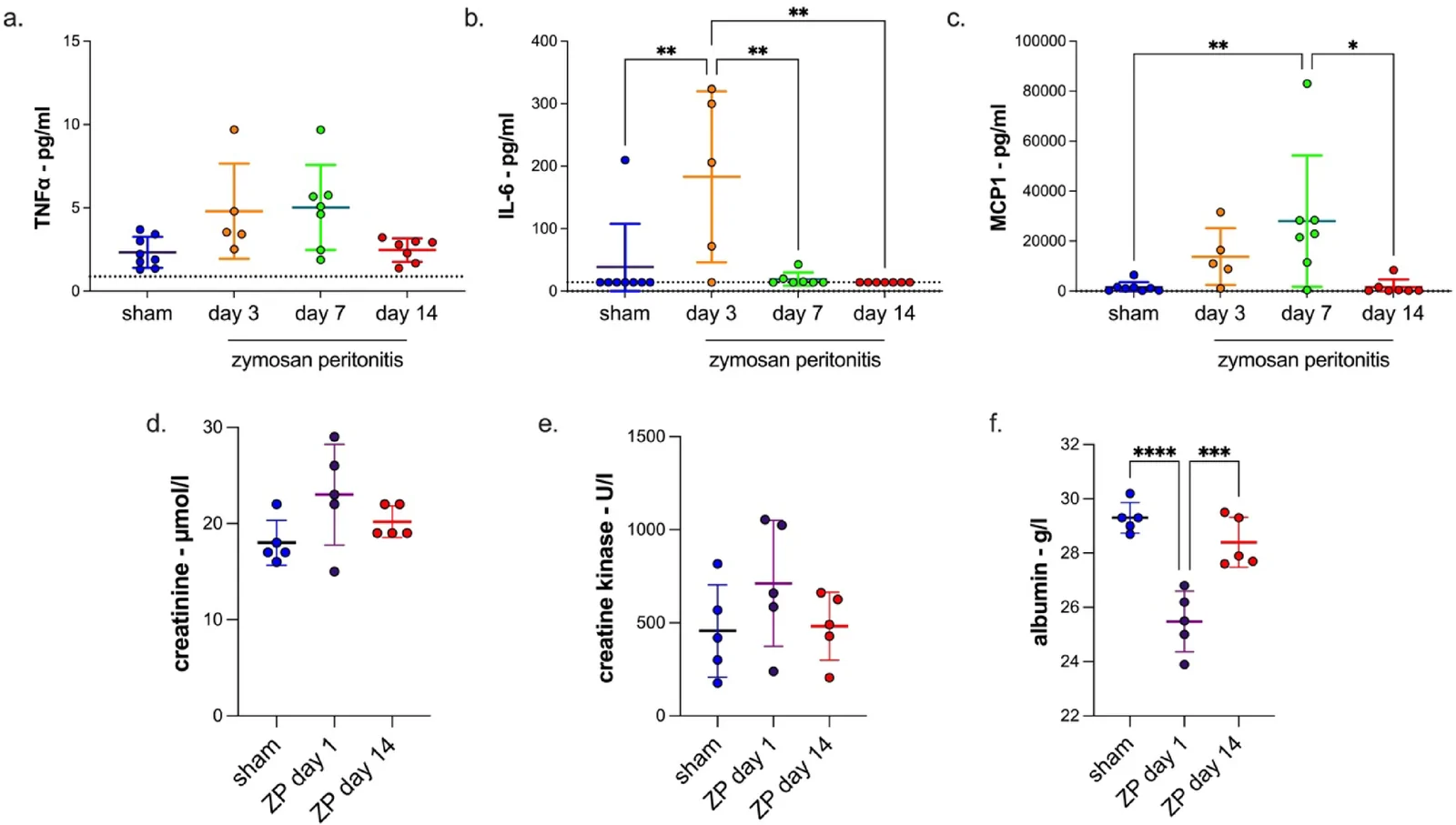
Circulating mediators of inflammation and organ function in animals recovering from zymosan peritonitis. TNFα, IL-6 and MCP1 levels were measured in serum from sham animals (n = 8) and animals at Days 3 (n = 5), 7 (n = 7) and 14 (n = 7) following zymosan peritonitis. (**a**) TNFα levels did not differ between sham and zymosan peritonitis groups. One way-ANOVA with Holm-Sidak multiple comparison test. (**b**) IL-6 was higher at zymosan peritonitis Day 3 compared to sham (*p* = 0.005), zymosan peritonitis Day 7 (*p* = 0.002) and Day 14 (*p* = 0.002). One way-ANOVA with Holm-Sidak multiple comparison test. (**c**) MCP1 was higher at zymosan peritonitis Day 7 compared to sham (*p* = 0.01) and day 14 (*p* = 0.01). One way-ANOVA with Holm-Sidak multiple comparison test. Circulating markers of organ function were measured in serum from sham animals (n = 5) and zymosan peritonitis animals at Day 1 (n = 5) and Day 14 (n = 5) following induction of zymosan peritonitis. (**d**) Creatinine did not significantly differ between groups. One way-ANOVA. (**e**) Creatine kinase did not differ significantly between groups. One way-ANOVA. (**f**) Albumin was significantly reduced at day 1 compared to sham (*p* < 0.0001) and zymosan peritonitis day 14 (*p* = 0.0005). One way-ANOVA.

Serum markers of organ function were measured in sham animals and at Days 1 and 14 of zymosan peritonitis. Urea was raised at Day 1 compared to Day 14 (*p* = 0.02) but creatinine levels were unchanged between groups. Alanine transaminase was significantly reduced at Days 1 and 14 compared to sham animals (both *p* < 0.01). Creatine kinase, a marker of muscle breakdown, was unchanged in animals with zymosan peritonitis compared to sham. Albumin, a negative acute phase protein, was reduced at Day 3 compared to sham (*p* < 0.0001) but had recovered by Day 14.

### Epididymal and retroperitoneal white adipose tissue do not demonstrate increased thermogenesis in the extended recovery phase of zymosan peritonitis (Fig. 3)

Thermal imaging of epididymal white adipose tissue (eWAT) was used to detect localized heat production. There was no significant difference in eWAT temperature (normalized to core temperature) between sham (n = 9) and zymosan peritonitis (n = 8) animals at day 14 (*p* = 0.37).

**Fig. 3:**
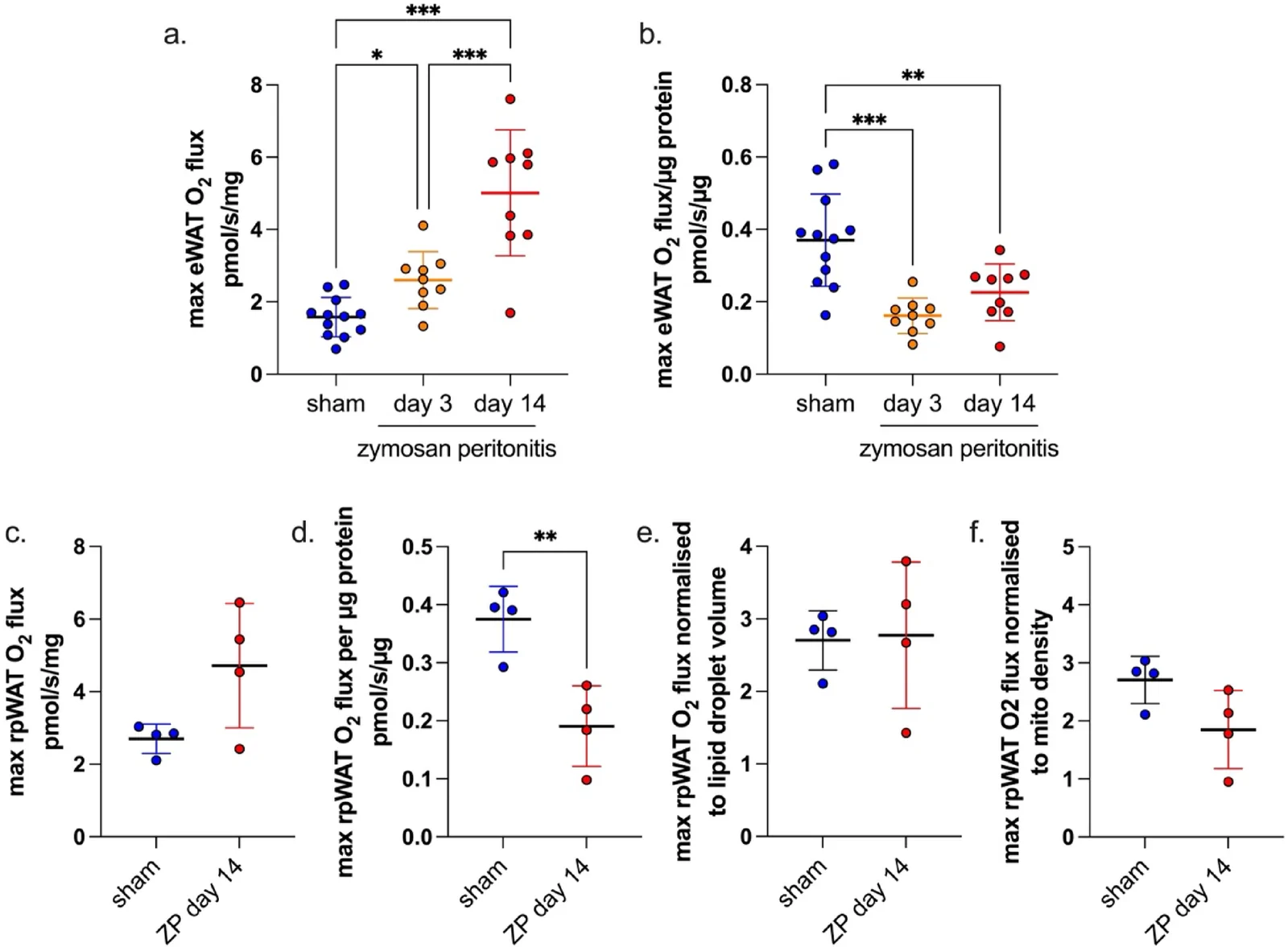
Maximum uncoupled total oxygen flux in epididymal and retroperitoneal white adipose tissue normalised to mass and protein content (eWAT and rpWAT) and cell number, lipid droplet volume and mitochondrial number (rpWAT only). Maximum uncoupled total oxygen flux was measured by high resolution respirometry in eWAT from sham animals and at Days 3 and 14 of zymosan peritonitis. Oxygen flux was normalized to tissue mass and protein content. (**a**) Oxygen flux per milligram of eWAT was significantly higher at zymosan peritonitis Day 3 compared to sham (*p* = 0.04) and at zymosan peritonitis Day 14 compared to sham (*p* < 0.001) and zymosan peritonitis Day 3 (*p* < 0.001). One way-ANOVA with Holm-Sidak multiple comparison test. (**b**) Compared to sham, oxygen flux per microgram of protein of eWAT was significantly lower at zymosan peritonitis Day 3 (*p* = 0.005) and Day 14 (*p* < 0.001). One way-ANOVA with Holm-Sidak multiple comparison test. Maximum uncoupled total oxygen flux was measured in rpWAT from sham animals and at Day 14 after zymosan peritonitis. Oxygen flux was normalized to tissue mass, tissue protein content, lipid droplet volume and mitochondrial density measured by multiphoton microscopy. (**c**) Compared to sham, maximum uncoupled total oxygen flux per µg rpWAT did not differ significantly at zymosan peritonitis Day 14, but did trend towards a higher rate (*p* = 0.06). Student’s t test. (**d**) Maximum uncoupled total oxygen flux of rpWAT per µg protein was lower at zymosan peritonitis Day 14 versus sham (*p* = 0.006). Student’s t test. (**e**) Maximum uncoupled total oxygen flux of rpWAT normalized to lipid droplet volume did not differ between tissues from sham animals and zymosan peritonitis Day 14 animals. Student’s t test. (**f**) Maximum uncoupled total oxygen flux of rpWAT normalized to mitochondrial density did not differ between tissues from sham animals and zymosan peritonitis Day 14 animals. Student’s t test.

Respirometry was performed on eWAT and rpWAT to measure maximum uncoupled total oxygen flux and identify thermogenesis in the extended recovery phase of zymosan peritonitis. Per milligram of tissue, maximum uncoupled total oxygen flux of eWAT was higher in zymosan peritonitis animals at Day 3 (2.3 (95% CI 2.10–2.50) vs. sham 1.70 (1.58–1.82) pmol/s/mg, *p* = 0.04), and Day 14 (5.0 (4.57–5.60) vs. sham 1.70 pmol/s/mg (1.58–1.82), *p* < 0.001). In zymosan peritonitis animals Day 14 eWAT maximum uncoupled total oxygen flux was significantly higher than at Day 3 (*p* < 0.001). This pattern was however reversed when oxygen consumption was controlled for by tissue protein concentration, with sham eWAT maximum uncoupled total oxygen flux being significantly higher at both Day 3 (0.37 (0.29–0.45) vs. zymosan peritonitis 0.16 (0.12–0.20) pmol/s/µg, *p* < 0.001) and Day 14 (0.37 (0.29–0.45) vs. zymosan peritonitis 0.23 (0.17–0.29) pmol/s/µg, *p* = 0.005). The reduction in oxygen consumption normalised to protein level may be explained by increased protein expression in the eWAT.

In rpWAT, maximum uncoupled total oxygen flux per milligram of tissue trended towards increased rates in zymosan peritonitis animals at Day 14 (4.7 (1.99–7.04) vs. sham 2.7 (2.05–3.35) pmol/s/mg, *p* = 0.06). This pattern was also reversed when controlled for by tissue protein concentration with sham maximum uncoupled total oxygen flux significantly higher at Day 14 (sham 0.38 (0.28–0.47) vs. zymosan peritonitis 0.19 (0.08–0.3) pmol/s/µg, *p* = 0.006).

Multiphoton microscopy interrogated changes in adipose tissue architecture and allowed normalization of tissue respirometry to cell number and mitochondrial density (detailed results below). When maximal rpWAT maximum uncoupled total oxygen flux was controlled for lipid droplet volume, cell number (measured by number of nuclei) and mitochondrial density (measured by multi-photon visualisation), there was no difference in maximum uncoupled total oxygen flux between sham and zymosan peritonitis adipose tissue, indicating the absence of a change to a thermogenic phenotype during recovery from zymosan peritonitis.

### The transcriptome of rpWAT taken from animals at day 14 of zymosan peritonitis indicates inflammation, downregulation of mitochondrial respiratory pathways and implicates adipose tissue as a mediator of cachexia, but does not support a switch to a thermogenic phenotype (Fig. 4)

The transcriptomes of bulk rpWAT from sham and Day 14 zymosan peritonitis rats were compared to identify differentially expressed genes. Day 14 of zymosan peritonitis was chosen as the animals had recovered from the acute illness yet still had a cachectic phenotype. RNA quality control was good and deemed adequate for downstream processing. Pearson correlation and principal component analysis showed separation between sham and zymosan peritonitis groups. Pearson’s correlates within groups were typically > 0.9. Inter-group comparison (e.g. sham samples 1–5 vs. sepsis samples 1–5) showed lower correlation coefficients, typically 0.6–0.8, indicating marked differences in gene expression between the two groups.

**Fig. 4:**
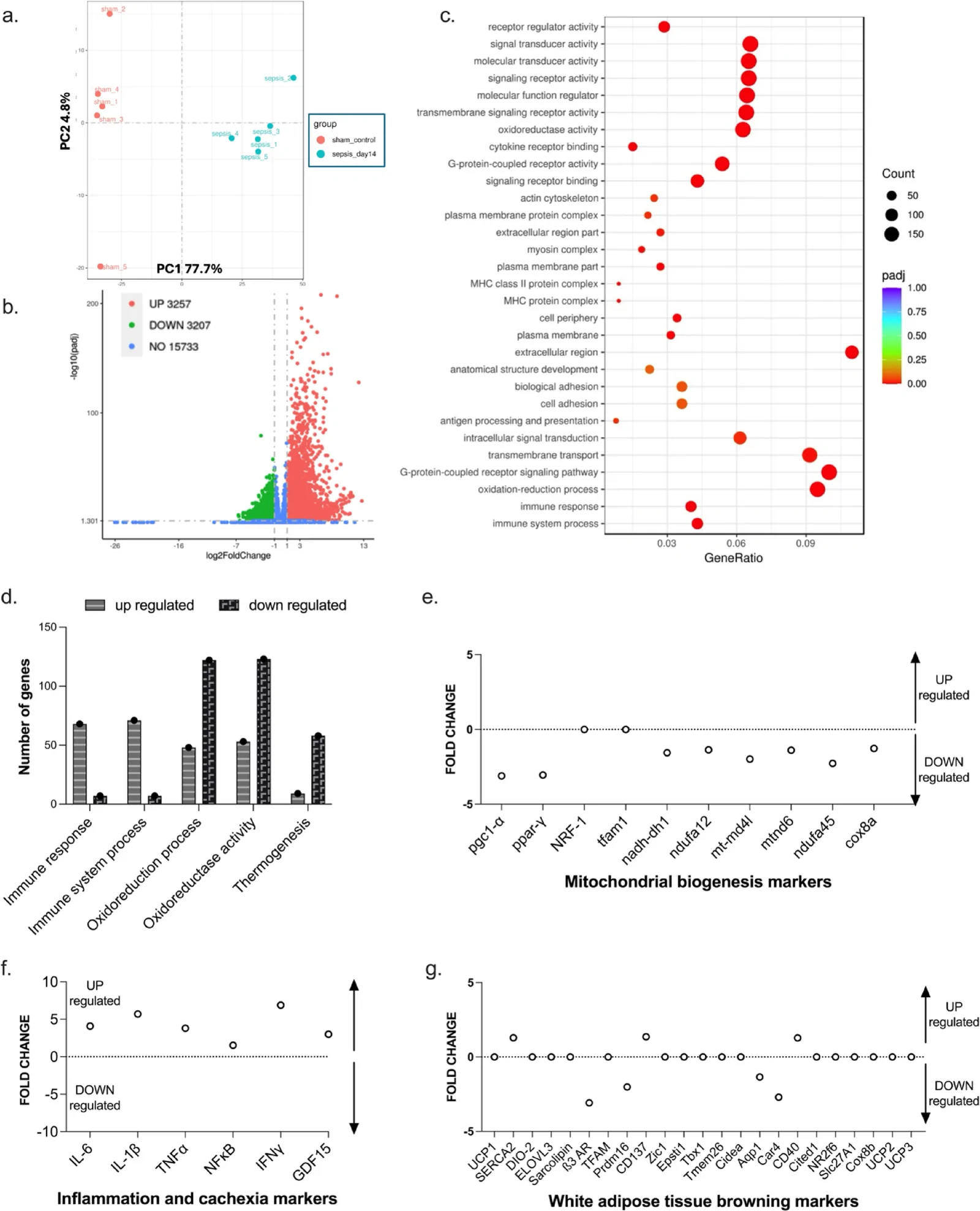
RNA sequencing of bulk rpWAT taken from 5 sham rats and 5 rats at Day 14 of zymosan peritonitis (named ‘sepsis’ in figure). (**a**) Principle Component Analysis (PCA) between samples showing strong intra-group concordance and inter-group separation. (**b**) Volcano plot of distribution of differentially expressed genes. Genes considered differentially expressed if log2 fold change > 1, and adjusted *p* value < 0.05). (**c**) Gene Ontology (GO) enrichment analysis displaying the 30 most significant GO terms. The ordinate (y-axis label) is the enrichment term, the abscissa (x-axis) is the GeneRatio calculated as the ratio of the number of differential genes linked with the pathway to the total number of differential genes. Point size indicated the number of genes in the pathway. Point colour indicated level of significance (*p* adjusted). Thermogenesis and browning are not supported. (**d**) Plot of significantly up- and down-regulated genes within gene enrichment terms related to thermogenesis. Genes considered differentially expressed if fold change ≥ 1 and adjusted *p* value < 0.05 following Benjamini and Hochberg correction for multiple hypothesis testing. (**e**) Individual gene markers related to mitochondrial biogenesis showed widespread down-regulation, indicating absence of mitochondrial biogenesis that is intrinsic to browning. (**f**) Select gene markers implicated in cachexia phenotypes. The presence of this inflammatory process is supported by upregulation of IL-6, Il-1β, TNFα and IFNγ. GDF15, a growth factor implicated in cancer cachexia, is elevated at Day 14 after zymosan peritonitis. (**g**) Expression of genes considered browning markers in rodents. Sixteen were unchanged, 4 down-regulated and 3 up-regulated.

The transcriptome of rpWAT from Day 14 of zymosan peritonitis showed down-regulation, up regulation and unchanged expression of pathways associated with oxidative phosphorylation and thermogenesis. However, these pathways were predominantly down-regulated, while there was an increase in inflammatory pathways. The largest and most significant increases in gene expression in the GO enrichment pathways were ‘Immune System Process’ and ‘Immune response’. KEGG enrichment echoed this with ‘cytokine–cytokine receptor interaction’ and ‘hemopoietic cell lineage’.

Of 23 classical browning markers, three were elevated, four decreased and 16 unchanged. mRNA expression of UCP1, the canonical marker of browning, was unchanged in animals recovering from zymosan peritonitis. SERCA2, CD137 and CD40 were increased (defined as a change in expression and an adjusted *p* value < 0.05). Expression of the ß_3_-adrenergic receptor, Aqp1, Car4 and Prdm16 were all decreased. Of note, SERCA2 expression was increased 1.3-fold (*p*_adj_ < 0.05).

Processes associated with thermogenesis and browning were also interrogated. Ten markers of mitochondrial biogenesis were assessed, of which eight were downregulated and two unchanged. Transcription of genes intrinsic to lipolysis—adipose tissue specific lipase (atgl) and hormone sensitive lipase (hsl)—were both downregulated.

Transcripts of the pro-inflammatory mediators IL-6, IL-1β, TNFα, NFκB and IFNγ were all elevated at Day 14 of zymosan peritonitis compared to sham.

Taken together, rpWAT did not express a thermogenic program at Day 14 during recovery from zymosan peritonitis, compared to sham tissue, but did show marked inflammation. Of note, expression of GDF15, a growth factor linked to cachexia in patients with cancer, was increased threefold (*p*_adj_ < 0.05)^34^.

### The canonical marker of browning, UCP1, is not expressed in adipose tissue in the recovery phase of zymosan peritonitis, but SERCA2 is (Fig. 5)

Protein expression of UCP1 in white adipose tissue is the canonical marker of browning^16^. Immunoblot for UCP1 protein was negative in eWAT from sham animals and at Days 3, 7 and 14 of zymosan peritonitis. Immunoblot for UCP1 was also negative in rpWAT from sham animals and at Day 14 of zymosan peritonitis. By contrast, sarcoplasmic endoreticulum calcium ATPase 2 (SERCA2) protein expression was increased 3.9-fold in rpWAT taken from zymosan peritonitis animals at Day 14 (*p* = 0.01).

**Fig. 5:**
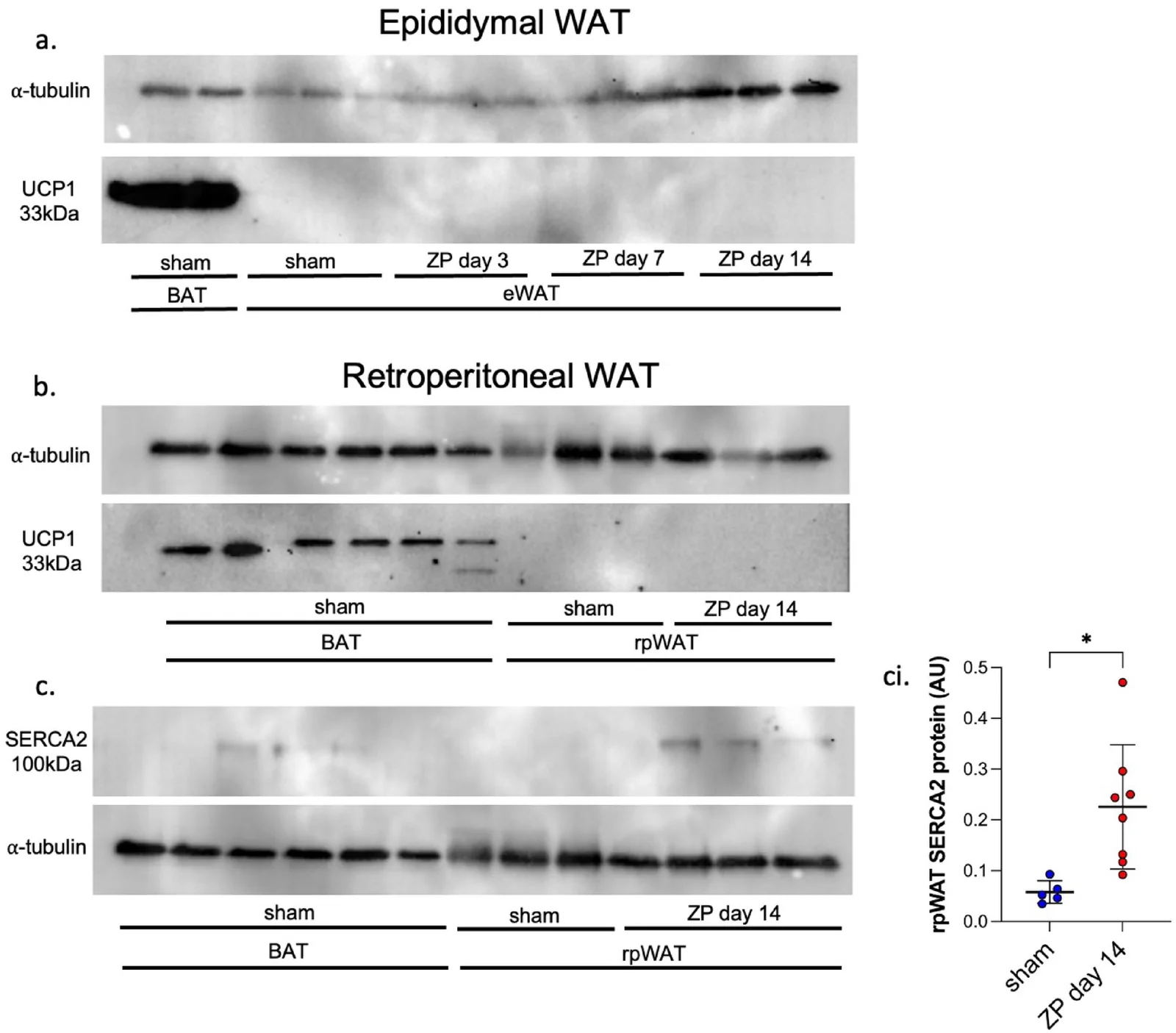
Protein expression of thermogenic mechanisms in eWAT and rpWAT from sham and septic animals. (**a**) Uncoupling protein-1 (UCP1) immunoblot. Brown adipose tissue (BAT) is the positive control. Epididymal white adipose tissue (eWAT) from sham animals and at zymosan peritonitis Day 14 showed no expression of UCP1 protein. (**b**) UCP1 immunoblot. BAT from sham animals is the positive control. rpWAT from sham animals and at zymosan peritonitis Day 14 of recovery showed no expression of UCP1 protein. (**c**) and (**ci**) Immunoblot of SERCA2 expression in BAT and rpWAT from sham animals (n = 5) and zymosan peritonitis Day 14 (n = 8). SERCA2 expression was elevated at zymosan peritonitis Day 14 compared to sham (*p* = 0.012).

### Multiphoton microscopy of rpWAT does not demonstrate morphological changes consistent with browning nor a thermogenic phenotype (Fig. 6)

Browning of white adipose tissue is associated with structural changes that enable thermogenesis, including lipid droplet loculation (smaller and more numerous lipid droplets) and increased mitochondrial density (through mitogenesis)^14^. We used multiphoton microscopy to interrogate the architecture of live explanted rpWAT for a thermogenic morphology. Lipid droplets in rpWAT from animals recovering from zymosan peritonitis (Day 14) had a significantly smaller volume (median 113,000 (95% (CI 140,000–154,000) µm^3^) compared to sham controls (192,000 µm^3^ (180,000–222,000), *p* < 0.0001). Virtual renderings of lipid droplets did not indicate multi-loculation, however, due to the resolution of the microscope, this could not be excluded. The ratio of lipid droplets to nuclei was calculated to identify immune cell infiltration and did not differ significantly between groups (*p* = 0.19), indicating no significant immune cell infiltration.

**Fig. 6:**
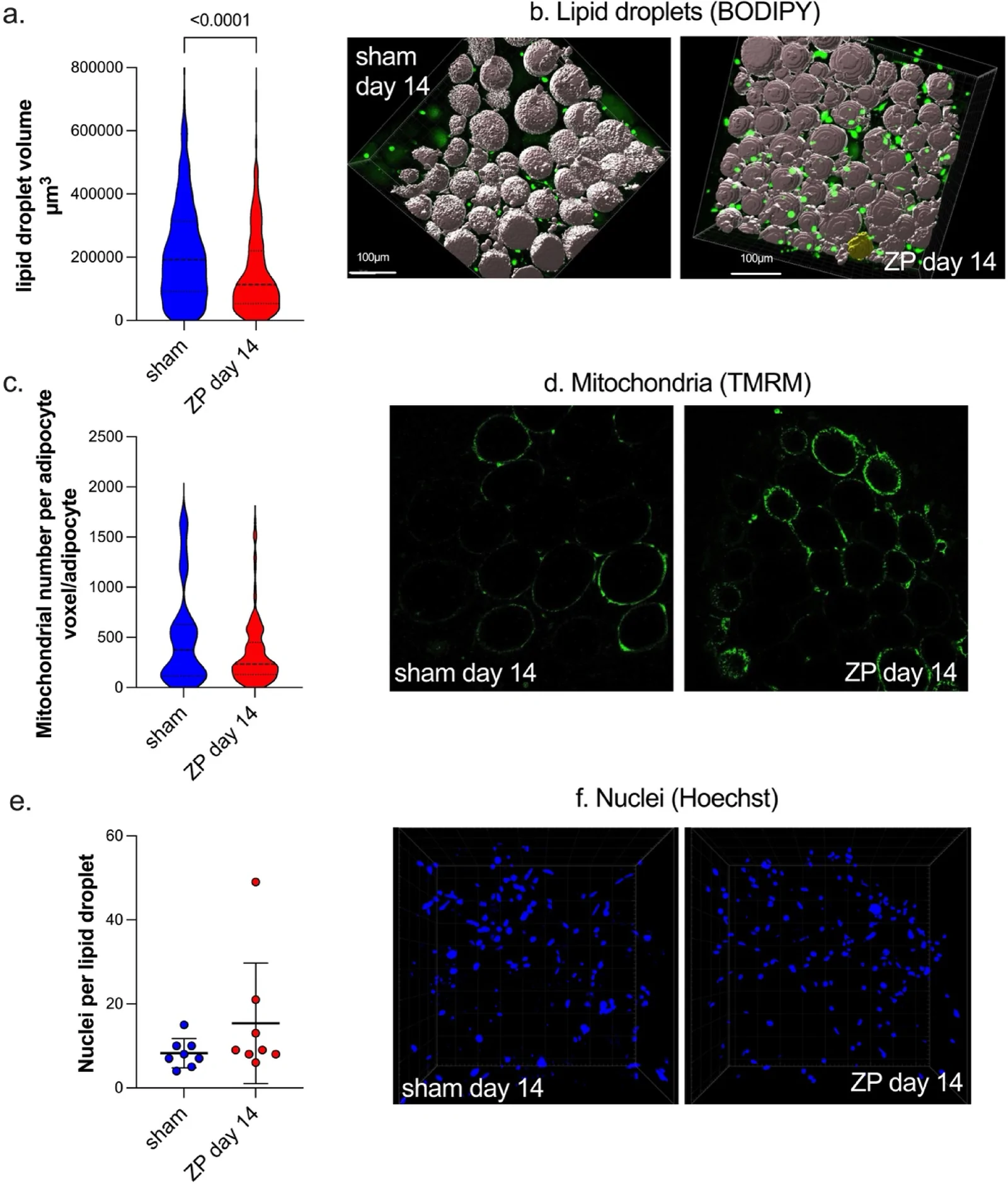
Morphology of retroperitoneal white adipose tissue at Day 14 of zymosan peritonitis: lipid droplet volume, mitochondrial density and cell number were measured by multiphoton microscopy in rpWAT from sham (n = 8) and zymosan peritonitis (n = 8) animals at day 14 after insult. (**a**) The impact of zymosan peritonitis on lipid droplet volume was measured following staining with BODIPY; lipid droplets showed a reduced volume in rpWAT taken from animals at day 14 of recovery from zymosan peritonitis (n = 8, mean number of events per sample = 510), compared to sham controls (n = 8, mean number of events per sample = 175). Mann–Whitney non-parametric test. (**b**) Representative 3D stack images of rpWAT stained with BODIPY for lipid droplets and Hoechst for nuclei (green) with surfaces generated over lipid droplets to allow measurement by Imaris software. (**c**) To measure mitochondrial biogenesis, mitochondrial density in rpWAT was measured in individual adipocytes after staining with TMRM. The number of mitochondria per adipocyte did not increase (sham = 8, mean number of events per sample = 499. Zymosan peritonitis = 8, mean number of events per sample = 156). (**d**) Representative images of rpWAT stained with TMRM to label mitochondria (yellow) in rpWAT from sham animals and at day 14 of recovery from zymosan peritonitis. (**e**) To quantify infiltration, nuclei were counted following staining of rpWAT with Hoechst. When normalized to the number of adipocytes (calculated as the number of lipid droplets) there was no significant difference. (**f**) Representative 3D stack images of rpWAT stained with Hoechst only, showing nuclei in rpWAT from sham animals and at zymosan peritonitis Day 14.

The number of mitochondria per imaging field was increased but the number per adipocyte was not increased (sham 373, Day 14 zymosan peritonitis 233 (95% CI of difference − 207 to 35, *p* = 0.24). The increase per imaging field is due to the reduction in lipid droplet volume, not an increase per adipocyte to indicate mitochondrial biogenesis.

## Discussion

Survivors of sepsis suffer muscle and tissue wasting long after discharge from intensive care^35^. Despite multiple abortive attempts, no effective treatment exists. The identification of browning in patients with burn injury- and cancer-associated cachexia and the increasing recognition of adipose tissue as a mediator of skeletal muscle wasting warrants further investigation of the role of adipose tissue in sepsis^10,12,34^. We sought to characterize browning in an experimental rat model of sepsis to build upon the sparse existing literature^26–28^. However, in contrast to previous studies, evidence of browning was absent at both transcriptomic, protein and functional studies in two adipose tissue deposits up to 14 days in this model.

The zymosan peritonitis model is well established and appropriate for the study of adipose tissue in the recovery phase of sepsis^30^. Although sterile, it mimics human sepsis by generating acute organ dysfunction consistent with a sepsis-like inflammatory illness, demonstrated here by a mortality rate of 17% and reduction in cardiac stroke volume at 6 and 24 h in animals receiving zymosan compared to sham (supplemental Fig. 1b). In survivors a hypermetabolic recovery phase is described causing both myopathy and cachexia^36,37^; these findings were reproduced in the present study.

Despite evidence of whole organism hypermetabolism, the WAT deposits studied were not contributory as they did not adopt either a thermogenic or hypermetabolic phenotype. To our knowledge, this is the first functional study of adipose tissue thermogenesis in experimental sepsis. When normalised to tissue wet weight, WAT maximum uncoupled total oxygen flux was increased, however, this is accounted for by an increase in the number of adipocytes in the sample caused by the reduction in volumes of lipid droplets. When normalised to methods aimed at controlling for the number of adipocytes, respirometry of eWAT and rpWAT showed either unchanged or reduced oxygen consumption during the extended recovery phase of zymosan peritonitis. This was reflected in the equivalent temperatures of eWAT on laparotomy at Day 14 as measured by thermal imaging. RNA-sequencing of bulk rpWAT did not support the presence of browning nor thermogenesis; indeed, while some genes related to oxidative phosphorylation, mitochondrial and thermogenic signatures were upregulated, on balance more were unchanged or downregulated, thereby not supporting a phenotypic switch to thermogenesis. Conclusively, the canonical marker of browning, UCP1, was absent in both eWAT and rpWAT. These findings consistently refute the presence of browning in this model, and open discussion on the likely presence of browning in other models and humans in the recovery phase of sepsis.

The reason for the absence of browning, in contrast to burn injury and cancer cachexia, is likely multifactorial. Burn injury is a profound form of injury. Browning is described in animals and humans suffering 30–60% total body surface area burns which results in a far greater and prolonged inflammation than that seen in sepsis^38,39^. Burn injury also perturbs thermostatic control through the loss of skin, hair and fluids and may drive cold stress, a known potent driver of browning^40^. In cancer patients, illness and inflammation generates a more protracted stimulus than that seen in sepsis^10^. It is plausible that the severity and length of a septic injury required to drive browning would be non-survivable.

Our study did reveal novel insights into adipose tissue physiology in the extended recovery phase of zymosan peritonitis. Sarcoplasmic endoreticulum calcium ATPase 2 expression was elevated at both mRNA and protein levels in rpWAT at 14 days. To our knowledge, this is a novel finding. SERCA2 is a calcium pump that actively transports calcium from cytoplasm into the sarco- or endo-plasmic reticulum (depending on cell type). It has many roles including adaptive thermogenesis via futile cycling in skeletal muscle and in brown and beige adipose tissue^17,41^. The absence of supportive mechanisms for thermogenesis (e.g. mitochondrial complexes) make this role unlikely. Its role here is currently unclear, but notably SERCA2 expression also increases during endoplasmic reticular stress and this is intrinsic to cachexia and adipose tissue dysfunction including hyperlipidemia, hepatic dysfunction and insulin resistance. This does suggest a role of adipose tissue as a metabolic mediator after critical illness^22^.

Persistent inflammation in adipose tissue following critical illness is well described^42,43^. It is likely to mediate prolonged critical illness and is intrinsic to insulin resistance and consequent hyperglycemia. It is also likely to play more roles than currently appreciated^22^. Indeed, a recent paper utilizing single cell transcriptomics showed that adipose tissue from mice recovering from sepsis remains more inflamed and perturbed than skeletal muscle a month after injury^42^. While our study did not focus on persistent inflammation, the transcriptomic data are supportive and in accord with the literature and support adipose tissue as a mediator and potential perpetuator of cachexia in sepsis; pro-inflammatory, cachexia-inducing cytokines TNFα and Il-6 mRNA were raised at 14 days, suggesting adipose tissue as a source of cachexia-inducing cytokines^44^.

Beyond classical mediators of inflammation and cachexia, GDF15, a highly conserved growth factor implicated in cancer-associated cachexia, also displayed increased mRNA expression at 14 days. GDF15 is not expressed in tissue in health but is associated with tissue wasting and anorexia during acute illness, including the acute phase of sepsis^45^. It is the most differentially and highly expressed plasma protein in cachetic lung cancer patients and is now touted as a putative therapeutic target^34^. This isolated finding requires further investigation but suggests adipose tissue may, in part, drive cachexia after sepsis.

### Limitations

Animal models can only approximate the complexity and heterogeneity of human sepsis. The zymosan peritonitis model is a single sterile insult leading to hyperinflammation, therefore does not precisely replicate sepsis due to the absence of infection. Our model also does not include pair feeding (to assess impact of reduced food intake) or potential iatrogenic sources of adipose tissue dysfunction such as catecholamine infusions and comorbidities such as obesity. Nor does the model include activity telemetry data to formalise the reduced activity seen in peritonitic animals which would impact whole organism respirometry data and potentially explain the reduced oxygen consumption in this group compared to sham animals. The study of cachexia is also complicated by the use of rats still in the growth phase; ideally aged rats would be used to more closely mimic human adult sepsis. Respirometry measurements on both eWAT and rpWAT did not include the addition of rotenone and antimycin A to identify non-mitochondrial respiration. Given the variable response to these inhibitors seen in the optimisation experiments (Supplemental Materials Fig. 3a) we cannot exclude that variation between sham and zymosan peritonitis samples could be due to unidentified non-mitochondrial respiration. The use of bulk tissue RNA sequencing may also dilute or obfuscate more nuanced changes in adipocyte and adipose tissue biology that may be more appreciable using single cell techniques. The absence of heterogeneity in the characteristics of the animal model, including sex, co-morbidity and age also limit reflection of human disease.

## Conclusion

In this model of experimental sepsis our data do not support the hypothesis of UCP1-dependent browning of adipose tissue as a driver for sepsis-induced cachexia. However, transcriptomic and protein level changes identified could indicate a role for adipose tissue in sepsis-induced cachexia through other mechanisms. Further study is warranted to explore these findings further.

## Supplementary Information

Below is the link to the electronic supplementary material.

Supplementary Material 1

## Data Availability

Data is provided within the manuscript or supplementary information file. The datasets generated and analysed during the current study are available in the NCBI Sequence Read Archive repository: [PRJNA1482020, https://www.ncbi.nlm.nih.gov/sra/PRJNA1482020].
