# Supplementary material for: Experimental sepsis causes SERCA2 expression in white adipose tissue but not classical browning
Source: Sci Rep. 2026 Jul 3;16:30713. doi: 10.1038/s41598-026-60904-0 (PMC13634910; doi:10.1038/s41598-026-60904-0)
Supplement: Supplementary file 1 [file 41598_2026_60904_MOESM1_ESM.docx]

**Supplemental Methods**

*Experimental sepsis animal model scoring system*

**Table 1: Validated scoring system of clinical illness for rats with sepsis** [1]

*Tissue permeabilisation experiments*

To assess the need for permeabilization, tissue from naive animals (n=3) was prepared as described above and placed in Oroboros chambers A and B (technical duplicates) without permeabilization agents. Saturating levels of succinate and ADP were then added. Any increase in oxygen flux indicates some degree of permeability. Rotenone was then added to block electron transport through complex I, ensuring all respiration occurs through complex II. The permeabilisation agent, digitonin, was then titrated against increases in oxygen flux, resulting in increased substrate availability. If no further increase was seen, cells were considered optimally permeabilized. In the absence of a permeabilization agent, any permeability can be attributed to mechanical permeabilization resulting from tissue handling and manipulation.[2]

A further experiment was conceived to ensure WAT was optimally permeabilized to succinate which is considered unable to cross intact cell membranes. WAT from three sham animals was prepared as above and added to the chambers in duplicate. Saturating levels of succinate were then added. Methyl-succinate, which is freely permeable through cell membranes, was then added to assess if further stimulation of complex II occurred, indicating incomplete permeabilization.

**Table 2. Constituents of Biopsy Preservation Media**

| **Constituent** | **Final concentration (mM)** |
| --- | --- |
| **CaK_2_EGTA** | 2.77 |
| **K_2_EGTA** | 7.23 |
| **Na_2_ATP** | 5.77 |
| **MgCl_2_•6H_2_O** | 6.56 |
| **Taurine** | 20 |
| **Na_2_Phosphocreatine** | 15 |
| **Imidazole** | 20 |
| **Dithiothreitol** | 0.5 |
| **MES hydrate** | 50 |
| **pH** | 7.1 |

*RNA sequencing*

mRNA was purified then fragmented and reverse transcribed to cDNA. cDNA underwent end-repair, A-tailing, adapter ligation, size selection, amplification and purification. Index coded samples were clustered using a cBot Cluster Generation System using TruSeq PE Cluster Kit v3 cBot HS and sequenced on an Illumina Novaseq platform. Post-sequencing quality control was performed to remove low quality reads from the data. The transcriptome was mapped to the rattus norvegicus reference genome using HiSat2 v2.0.5. Mapped reads were then assembled using StringTie v1.3.3b to quantitate full length transcripts for each gene locus. Gene expression estimates were calculated as reads mapped to each gene and expressed as Fragments Per Kilobase of transcript sequence per Million base pairs sequenced (FPKM), which accounts for gene length and sequencing depth.

Differential gene expression between sham and sepsis recovery rpWAT was performed using SESeq2 R package 1.20.0. P-values were adjusted (padj) using Benjamini and Hochberg’s correction to control for false positives as a result of multiple testing. Adjusted p-values ≤0.05 were considered significant. Gene Ontology (GO) and Kyoto Encyclopaedia of Genes and Genomes (KEGG) enrichment analysis was performed to cluster biologically related genes and identify differentially expressed pathways. Paired-end sequencing to a length of 150 base pairs was performed to generate high-quality alignable sequence data. Read length of 150 base pairs was chosen as sufficient for accurate expression profiling.

*Western Blot*

Western blot was performed to compare protein expression between sham control tissues and tissues from animals recovering from sepsis.

*Western blot sample preparation*

50-100mg white adipose tissue was immersed in ice-cold radio-immunoprecipitation assay buffer containing protease and phosphatase inhibitors (Pierce, Thermo Scientific, Massacheusetts, USA). Tissue was homogenised by bead milling in a Minilys Homogenizer (Bertin Technologies, Ile-de-France, France) for 20 seconds and repeated twice, with intervals of at least 60 seconds on ice between rounds. Samples were then left on ice for 30 minutes before centrifugation at x12000 rpm at 4ºC for 10 minutes. This produces a three-layered product: the bottom layer of cell debris combined with beads, the middle liquid supernatant containing proteins of interest, and a dense fatty ‘cake’ at the surface. To avoid contamination of the liquid supernatant with fat, a 21-gauge needle was introduced through the side of the bead-milling tube below the fat cake and the liquid supernatant aspirated into a fresh 1.5 ml Eppendorf tube. The process was repeated twice to minimise fatty contamination of the sample.

Lysate protein concentration was calculated by a bicinchoninic (BCA) protein assay (Pierce, Thermofisher Scientific, Massacheusetts, USA) with samples diluted in 5x loading buffer and RIPA to ensure 40 µg of protein per 14 µl.

*Electrophoreisis, transfer and development*

Electrophoresis was performed using hand-cast 10% SDS-Page gels with 15 wells per gel using a Bio-Rad mini-Protean chamber. 14 µl of sample were added per well. Gels were run at 120V until loading buffer reached the end of the gel. Gel contents were then transferred onto PVDF membrane (Immobilon-P, Merck New Jersey, USA) using a Bio-rad Trans-Blot semi-dry transfer cell.

Membranes were washed in TBST and stained with ink to check for uniform protein transfer before blocking with 5% BSA in TBST for 1 hour at room temperature. Membranes were incubated with the primary antibody overnight at 4ºC (see Tables 5 and 6 for primary and secondary antibodies). Membranes were incubated with appropriate secondary antibodies for an hour at room temperature. Blots were then developed by chemiluminescence onto film or using a Biorad ChemiDoc system (Bio-Rad, UK).

All blots were first developed for the protein of interest (UCP1 and SERCA2) and then stripped and re-stained to measure reference proteins (alpha-tubulin) to avoid cutting the membrane and investigate staining across the full range of protein sizes. Membranes were washed in stripping buffer for 10 minutes at room temperature three times then washed in PBS for 10 minutes twice then washed in TBST for 10 minutes three times. Blocking was then performed as described above.

**Table 2. Table of primary antibodies used for Western blots.**

All diluted in 5% BSA in TBST with 0.02% sodium azide.

| **Target protein** | **Primary Antibody** | **Dilution** | **Blocking** |
| --- | --- | --- | --- |
| UCP1 | Santa Cruz 293418 | 1/1000 | 5% BSA |
| SERCA2 | Santa Cruz 376235 | 1/500 | 5% BSA |
| α-tubulin | Cell Signalling Tech 2125S | 1/3000 | 5% BSA |

**Table 3. Table of secondary antibodies used for Western blot.**

All diluted in 5% BSA in TBST without sodium azide.

| **Target species** | **Secondary Antibody** | **Dilution** |
| --- | --- | --- |
| Rabbit | Polyclonal goat P0448 | 1/3000 |
| Mouse | Polyclonal rabbit P0260 | 1/3000 |

*Multiphoton microscopy of live rpWAT explants*

Tissue was mounted in a 35mm transparent dish within a Petri dish acting as a gutter. Tissue was anchored under a net fashioned from a stainless steel washer and fishnet-tight material to prevent floating and tissue drift and allowing the water immersion objective to dip into imaging buffer (physiological saline with HEPES). Bright-field imaging was then used to identify and focus on adipocytes before fluorescence imaging.

*Image acquisition - Adipose tissue architecture*

To determine lipid droplet volume and loculation and cell number, rpWAT was incubated in PSS+HEPES containing 10µM BODIPY (lipid stain) and 10ng/ml Hoechst (nuclear stain) for 10 minutes at 37ºC before imaging. A stack encompassing an entire layer of adipocytes (typically 100µm) in 5µm slices. See table for imaging set up.

*Image acquisition - Mitochondrial density*

rpWAT was incubated in PSS+HEPES containing 50nM fluorophore TMRM for 20 minutes at 37ºC before imaging. The concentration and time were optimised for stable staining. A 100µm stack of 5µm slices was taken to encompass multiple adipocytes.

*Image Analysis - Adipose tissue architecture*

Lipid droplet volume was measured using Imaris Software (Oxford, UK). Objects within the green channel capable of containing a 35µm sphere were selected. Surfaces were then generated for each lipid droplet and the threshold altered to remove artefact and debris. Droplets were checked to ensure separation and ‘cut’ if falsely joined.

Nuclear number was also measured using Imaris software. Objects in the blue channel capable of containing a 5µm^3^ sphere were selected. The threshold was then altered to ensure object selection was specific to nuclei.

Mitochondrial density was measured using Fiji (Image J v2.3.0/1.53q) by converting 3D stacks into a 2D SumSlices Z project and thresholded using the Otsu algorithm. Individual adipocytes were then isolated using a region of interest and positive pixels taken to represent mitochondria. Density was then calculated as the proportion of positive pixels in the total adipocyte area. To calculate the number of mitochondria per cell, the mitochondrial density was normalised to the number of lipid droplets as adipocytes visualised had one droplet and therefore represented the number of adipocytes.

**Table 4. Microscopy settings for live rpWAT explant imaging using multiphoton microscopy.**

|  | **Mitochondria** | **rpWAT architecture** |
| --- | --- | --- |
| **Size** | 1024x1024 | 1024x1024 |
| **Laser wavelength** | 800nm | 800nm |
| **Laser power** | 10% | 40% |
| **Master gain** | 50% | 50% |
| **Smart gain** | 150 | 500 |
| **Offset** | 50 | 50 |
| **Objective** | 25x 0.95 NA | 25x 0.95 NA |
| **Line averaging** | 2 | 2 |
| **Emission spectra** | 550-610nm TMRM | C1 – 450-490nm Hoechst  C2 – 490-530nm BODIPY |
| **Pinhole** | 10 airy units | 10 airy units |
| **Digital zoom** | 1 | 1 |
| **Stack depth** | 100µm | 100µm­+ |
| **Slice depth** | 5µm | 5µm |
| **Timelapse** | 1 stack | 1 stack |
| **Temperature** | 37ºC | 37ºC |
| **Pharmacological manipulation** | Nil | Nil |

**Table 5. Constituents of physiological saline solution used for live tissue explant imaging.**

| **Constituent** | **Final concentration (mM)** |
| --- | --- |
| **NaCl** | 118 |
| **KCl** | 4.7 |
| **NaHCO_3_** | 10 |
| **MgSO_4_** | 1.44 |
| **KHPO_4_** | 1.2 |
| **CaCl** | 1.8 |
| **Glucose** | 5 |
| **Pyruvate** | 5 |
| **HEPES** | 10 |
| **pH** | 7.4 |

**Table 6. Aide to understanding experimental timepoints.** Colour filled boxes indicate the day(s) of the measurement.

**Supplemental Results**

d.

**Supplement to Figure 1. Physiological parameters of the 14-day rat zymosan peritonitis model of experimental sepsis.**

a) Clinical illness severity score assessed on appearance, behaviour and clinical status (scores 1-3) was significantly higher in zymosan peritonitis versus sham rats (AUC 5.9 (CI 4.2-7.6) vs 0, p<0.05) with peak illness present at days 1-2 and recovery by day 7.
b) Stroke volume (SV) measured by echocardiography in 6 sham and 9 zymosan peritonitis rats at baseline, 6, 24, 48, 72 hours and 7 days. SV was significantly reduced at 6 (***p<0.001) and 24 hours (*p<0.05). Two way-ANOVA with post-hoc analysis.
c) Core temperature measured by colonic probe inserted under anaesthesia at baseline, 6 and 24 hours and at 3, 7 and 14 days. Temperature did not differ between groups. Two way-ANOVA with post-hoc analysis.
d) VCO_2_ production was reduced in animals with sepsis from days 0-8 compared to sham animals. From days 8-14, CO_2_ production was equivalent between groups.

**Supplement to Figure 2. Circulating markers of organ function.**

a) Urea was lower at zymosan peritonitis Day 14 compared to Day 1 (p=0.02). One way-ANOVA.
b) Compared to sham, alanine transaminase was lower at zymosan peritonitis Day 1 (p<0.0001) and Day (p=0.007). One way-ANOVA.

**Supplement to Figure 3. Optimisation experiments to establish white adipocyte permeability during high resolution respirometry.**

a) Standard substrate-uncoupler-inhibitor trial (SUIT) was used to interrogate adipocyte respiration. Baseline respiration was measured before addition of electron donors to complex I (PMG – pyruvate, malate, glutamate) followed by addition of succinate (Succ) as an electron donor to complex II. FCCP was then added as a chemical uncoupler to establish maximum uncoupled total oxygen flux Rotenone and Antimycin A were then added to establish non-mitochondrial oxygen consumption (not done in later experiments).
b) Digitonin optimisation protocol demonstrated adipocytes were adequately permeabilized and addition of permeabilisation agent, digitonin, did not increase permeability substrates.
c) Addition of freely permeable methylsuccinate (CH_3_-Succ) did not increase oxygen consumption, indicating that the adipocytes are fully permeabilized to saturating levels of succinate.

**Supplement to Figure 3. Functional measurement of thermogenesis in adipose tissue at**

**day 14 of recovery from zymosan peritonitis**

a) Oxygen flux of rpWAT normalized to number of nuclei was did not differ between sham animals and zymosan peritonitis Day 14. Student’s t test.
b) eWAT temperature measured by thermal imaging and normalized to core temperature did not differ between groups. Student’s t test.
c) Representative thermal imaging pictures of laparotomized rats at zymosan peritonitis Day 14. eWAT was identified by a needle. Boxes 1 and 2 are areas used to measure eWAT and liver temperature (measured for alternative normalisation method – data not shown), respectively.

**Supplement to Figure 5. Uncropped immunoblots of protein expression of thermogenic**

**mechanisms in eWAT and rpWAT from sham and septic animals.** Please see the main text

for experimental details. Of note, the large water mark across the blots is caused by an

issue with the ChemiDoc, as demonstrated by its consistency across blots. A single blot is shown for eWAT alpha-tubulin and UCP1 as stripping of UCP1 was unsuccessful, so both proteins are shown simultaneously.

**Supplement to Figure 6. Morphology of retroperitoneal white adipose tissue at Day 14 of zymosan peritonitis**

a) To measure mitochondrial biogenesis, mitochondrial density in rpWAT was measured in individual adipocytes after staining with TMRM. The density of mitochondria was increased in rpWAT from zymosan peritonitis Day 14 animals but the total number per adipocyte did not increase because of overall shrinkage of the lipid droplet and therefore adipocytes.
b) To quantify infiltration, nuclei were counted following staining of rpWAT with Hoechst. There were significantly more nuclei per imaging field in rpWAT from animals recovering from zymosan peritonitis, compared to sham (432 vs 250, p=0.02). Unpaired Student's t test. When normalized to the number of adipocytes (calculated as the number of lipid droplets), there was no significant difference.

Supplemental references:

1.	Brealey D, Karyampudi S, Jacques TS, Novelli M, Taylor V, Smolenski RT *et al.* Mitochondrial dysfunction in a long-term rodent model of sepsis and organ failure Mitochondrial dysfunction in a long-term rodent model of sepsis and organ failure. 2011;491–497.

2.	Romeiro NC, Ferreira CM, Oliveira MF. Assessment of mitochondrial physiology of murine white adipose tissue by mechanical permeabilization and lipid depletion. *Anal Biochem* 2020;611.
